# Warmer Environments Increase Implicit Mental Workload Even If Learning Efficiency Is Enhanced

**DOI:** 10.3389/fpsyg.2020.00568

**Published:** 2020-04-01

**Authors:** Tsukasa Kimura, Noriko Takemura, Yuta Nakashima, Hirokazu Kobori, Hajime Nagahara, Masayuki Numao, Kazumitsu Shinohara

**Affiliations:** ^1^The Institute of Scientific and Industrial Research, Osaka University, Ibaraki, Japan; ^2^Institute for Datability Science, Osaka University, Suita, Japan; ^3^Daikin Industries, Ltd., Osaka, Japan; ^4^Graduate School of Human Sciences, Osaka University, Suita, Japan

**Keywords:** thermal environment, learning efficiency, mental workload, EEG, autonomic nervous system

## Abstract

Climate change is one of the most important issues for humanity. To defuse this problem, it is considered necessary to improve energy efficiency, make energy sources cleaner, and reduce energy consumption in urban areas. The Japanese government has recommended an air conditioner setting of 28°C in summer and 20°C in winter since 2005. The aim of this setting is to save energy by keeping room temperatures constant. However, it is unclear whether this is an appropriate temperature for workers and students. This study examined whether thermal environments influence task performance over time. To examine whether the relationship between task performance and thermal environments influences the psychological states of participants, we recorded their subjective rating of mental workload along with their working memory score, electroencephalogram (EEG), heart rate variability, skin conductance level (SCL), and tympanum temperature during the task and compared the results among different conditions. In this experiment, participants were asked to read some texts and answer questions related to those texts. Room temperature (18, 22, 25, or 29°C) and humidity (50%) were manipulated during the task and participants performed the task at these temperatures. The results of this study showed that the temporal cost of task and theta power of EEG, which is an index for concentration, decreased over time. However, subjective mental workload increased with time. Moreover, the low frequency to high frequency ratio and SCL increased with time and heat (25 and 29°C). These results suggest that mental workload, especially implicit mental workload, increases in warmer environments, even if learning efficiency is facilitated. This study indicates integrated evidence for relationships among task performance, psychological state, and thermal environment by analyzing behavioral, subjective, and physiological indexes multidirectionally.

## Introduction

Climate change is one of the most important issues for humanity. This problem influences not only environmental destruction and pollution but also infrastructure such as water and energy, health, production of food, and economic activity (e.g., Haines and Ebi, 2019). To defuse this problem, it is considered necessary to improve energy efficiency, make energy sources cleaner, and reduce energy consumption in urban areas (e.g., Field et al., 2014). To reduce the consumption of energy, various efforts are being made in Japan. According to a government report in 2017, 25% of the energy consumption in Japan occurs in offices and homes (Agency for Neural Resources and Energy, 2017). Therefore, energy conservation is also recommended in offices and homes in Japan. As part of the home and office conservation effort, fixed settings are recommended for room temperatures controlled by air conditioners. For example, the Japanese government has recommended an air conditioner setting of 28°C in summer and 20°C in winter since 2005. These settings, called “COOLBIZ” and “WARMBIZ,” were decided according to the higher and lower limit to invoke cooling, set by the “Act on Maintenance of Sanitation in Buildings” (Ministry of Health Labour and Welfare, 1970). The aim was to save energy by keeping room temperatures constant. Even if “COOLBIZ” and “WARMBIZ” have been widely accepted in Japan so far, it is unclear whether they create an appropriate temperature for workers and students.

Environmental psychology studies have reported that a room’s temperature influences cognitive performance (see a review by Pilcher et al., 2002; Taylor et al., 2016). For example, reaction time (RT; e.g., Gaoua et al., 2012), memory (e.g., Racinais et al., 2008), and working memory (e.g., Gaoua et al., 2011) were impaired by hot and cold room temperatures. Moreover, performance of office work (e.g., Niemelä et al., 2002; Tanabe et al., 2009) and study in school (e.g., Haverinen-Shaughnessy and Shaughnessy, 2015; Wargocki et al., 2019), which are thought to use such cognitive functions, were also impaired. These environmental psychology studies suggest that room temperature influences not only limited cognitive function but also a wide range of cognitive functions related to daily life. In addition, experimental psychology studies have reported that physical and cognitive performance changes over time. For example, sustained attention decreases over time spent on a task. Moreover, this decrease makes the processing of a stimulus less active (e.g., Bonnefond et al., 2010), and requires more cognitive resources to process it (e.g., Smit et al., 2004). Putting these studies together, it is possible that a room’s temperature influences performance, which changes with time. Previous studies (Tanabe et al., 2009, 2015) reported that the fixed room temperature setting in Japan (i.e., 28°C in summer and 20°C in winter) might cause adverse effects on work and study. These studies showed that the amount of work performed in the office decreases every time the room temperature increases by 1°C, even if the room temperature is under 28°C (Tanabe et al., 2009) and the use of cooling is necessary to improve thermal comfort and the amount of work performed in the office when the room temperature is 28°C (Tanabe et al., 2015). Work time and energy consumption increase if the fixed temperature setting impairs workers’ and students’ efficiency. In other words, it is possible that this setting causes the opposite of energy conservation. It is necessary to examine the relationship between fixed temperature settings and work and study in order to avoid such a problem.

For this purpose, we developed a learning task that can measure change in performance over time and manipulated the room temperature setting. First, participants were asked to read a displayed text at their own pace and memorize the details of this text. Next, they were required to respond to a word presented on the display, stating whether the word existed in the text or not. In this task, learning efficiency was measured by time spent reading the text as the input efficiency for information, RT, and hit rate of words as the output efficiency for information. High learning efficiency should be based on efficient processing of working memory, which temporarily stores and processes information and passes information to and from long-term memory. In fact, working memory, which temporarily stores and processes information and passes information to and from long-term memory, is related to various cognitive processes (e.g., Baddeley, 2000) and should be closely related to learning efficiency. Therefore, we evaluated performance of working memory in terms of information input (i.e., time needed to read texts) and output (i.e., RT and hit rate for displayed words).

Moreover, we examined whether this performance changed over time when doing the task repeatedly. Previous studies reported that the performance of tasks that required attention decreased over time (e.g., Smit et al., 2004; Bonnefond et al., 2010). Therefore, we predicted that if learning efficiency decreases over time, reading time for the texts, and RT for the words would be longer and hit rate would decrease gradually. Furthermore, the room temperature was fixed at 18, 22, 25, or 29°C and room humidity was fixed at 50% during this task to examine the effect of the fixed room temperature on learning efficiency. Previous studies reported that fixed settings of room temperatures in Japan might cause adverse effects on work and study (Tanabe et al., 2009, 2015). Therefore, we predicted that if the fixed setting of room temperature causes adverse effects on work and study, at 18 and 29°C which are mostly the same as the fixed settings in Japan, reading time for the texts and RT for the words would be longer and hit rate would decrease. Furthermore, the working memory score was measured to examine the transition of temporarily stored and processed information (Siedlecki, 2007; Sliwinski et al., 2018). We predicted that performance of working memory, which was evaluated by the working memory score, would decrease at 18 and 29°C compared with the other temperature setting.

In addition, we examined whether the relationship between the fixed setting of room temperature and performance of learning efficiency influences psychological states. To examine this, we recorded electroencephalogram (EEG), heart rate variability (HRV), skin conductance level (SCL), and tympanum temperature during the task. The theta (4–7 Hz) power of the EEG was analyzed as an index of concentration and mental workload. Previous studies reported that this EEG recorded by electrode in the frontal region reflects cognitive control, and that the power increases with task difficulty and mental effort (e.g., Ishihara and Yoshii, 1972; Gevins et al., 1997; Borghini et al., 2014; Maurer et al., 2015). In particular, a concentration-demanding task such as an n-back task activates this power (e.g., Ishii et al., 1999). Moreover, previous studies reported that this power has been found to positively correlate with the level of workload (e.g., Miklody et al., 2017; Di Flumeri et al., 2018). Therefore, we predicted that if the fixed setting of room temperature impedes the learning task, more concentration and mental workload would be required for the task, and the theta power would increase. Moreover, the HRV was analyzed as an index of mental workload. The heart is influenced by parasympathetic and sympathetic activity, and the inter-beat interval (IBI) is changed by this activity. A low frequency component (LF; 0.04–0.15 Hz), which reflects the parasympathetic and sympathetic activity and a high frequency component (HF; 0.15–0.4 Hz), which reflects the parasympathetic activity can be calculated by conducting a frequency analysis of this IBI (e.g., Katona and Jih, 1975; Grossman and Svebak, 1987). Additionally, it is possible to examine which is the dominant of the parasympathetic and sympathetic activity by analyzing the ratio of LF to HF (LF/HF). The LF/HF ratio can be used as an index of mental workload, and it is known that this value increases with greater mental workload (e.g., Berntson and Cacioppo, 2004). Furthermore, the SCL was analyzed as an index of mental workload. Sweating is facilitated via sympathetic activity caused by mental or physical stress; this sweating is called “mental sweating.” It is possible to measure mental sweating as skin resistance by putting electrodes on the skin and applying a weak voltage. The SCL is the inverse of skin resistance and reflects sympathetic activity and increases generally with arousal (e.g., Dawson et al., 2007; Boucsein et al., 2012). Moreover, previous studies reported that the SCL reflects mental workload, i.e., mental stress (e.g., Reimer and Mehler, 2011; Foy and Chapman, 2018). Therefore, we predicted that if the fixed temperature setting impedes the learning task, mental workload would increase for the task, and the LF/HF and SCL would increase. In addition, tympanum temperature was also measured to examine the relationship between the room temperature and the body temperature of the participants.

Finally, subjective ratings for mental workload were measured by arousal and valence (Russell, 1980), and NASA-TLX in a Japanese version (Hart and Staveland, 1988; Haga and Mizukami, 1996). We predicted that if the fixed temperature setting impedes the learning task, subjective mental workload would increase for the task, and these rating scores would increase.

## Materials and Methods

### Participants

Twenty-four undergraduate and graduate students (11 females, 13 males; 20–24 years of age) participated in the experiment. One participant was left-handed and the others were right-handed, according to their self-report. All participants had normal or corrected-to-normal vision. This experiment was approved by the Behavioral Research Ethics Committee of the Osaka University School of Human Sciences. Written informed consent was obtained from all participants, and their rights as experimental subjects were protected.

### Stimulus and Equipment

#### Learning Task

Sixty texts extracted from web news, paperbacks, and Japan government reports were used as the learning stimuli. These texts were constructed by 1,600–1,800 characters per text. In this study, the visual angle of one character was 1.00° × 1.00° from a viewing distance of 60 cm (i.e., 28 points in terms of Microsoft office). In addition, 12 words extracted from these texts and other texts were used as the memory stimuli. These words were classified by frequency of appearance and co-occurrence frequencies as keywords (three words per one text), non-keywords (three words per one text), and novel words (six words per one text). Keywords had a HF of appearance and high co-occurrence frequencies, non-keywords had low frequencies, and novel words did not appear in the text.

#### Working Memory Task

The working memory score was measured by red dots in a five-by-five black grid. In addition, six-by-five letters (E and F) were used as distraction stimuli (Siedlecki, 2007; Sliwinski et al., 2018).

#### Subjective Rating

Arousal and valence (Russell, 1980) were measured on a 9-point scale (scores range from 1 to 9). A low score meant low arousal and unpleasantness. Moreover, five items from the Japanese version of NASA-TLX (Hart and Staveland, 1988; Haga and Mizukami, 1996) were measured on a 10-point scale (scores range from 0 to 9) and consisted of mental demand, performance, effort, frustration, and overall mental workload. A low score meant low mental demand, low effort, low frustration, and low mental workload. A low score only in performance meant high performance, because it was an inverted scale. Furthermore, thermal sensation and humidity sensation were measured on a 7-point scale (scores range from 1 to 7). A low score meant cold and dry.

#### Equipment

The presentation of stimuli, scales, and instructions were controlled with MATLAB R2010b (MathWorks, Inc.) and Psychtoolbox (Kleiner et al., 2007) installed on a desktop computer (STYLE-R027-i7-HN, iiyama). In addition, a 21.5-inch LCD monitor (XU2290HS-B2, iiyama) was put on the desk to present these stimuli, scales, and instructions.

#### Recording of Psychophysiological Data

Electroencephalogram data were recorded by Polymate mini AP108 (Miyuki Giken) and an electrode cap (g.tec medical engineering) using gold plated electrodes (Miyuki Giken) at eight sites (Fp1, Fp2, F7, F3, F4, F8, T7, and T8) according to the modified 10–20 System. In addition, electrodes were placed on both earlobes (A1 and A2), and these electrodes were used as the reference and the ground electrode. The data from all channels were recorded using the Mobile Acquisition Monitor Program (Miyuki Giken). The electrode impedances were kept below 300 kΩ. A DC filter was used at recording. The sampling rate was 500 Hz.

Electrocardiogram (ECG) data were recorded by eight-channel amplifier BA2008 (Miyuki Giken) using disposable electrodes (Mets Inc.). These electrodes were put on three sites according to the modified Lead II. SCL data were recorded by EDA amplifier MaP1720CA and EDA unit AP-U030 (Nihonsanteku) using disposable electrode (Mets Inc.). These electrodes were put on the left index and middle finger. Tympanum temperature data were recorded by temperature adaptor AP-U019 and sensor AP-C052m (Miyuki Giken). These autonomic nervous system data were recorded using InputMonitor software (Nihonsanteku), and the sampling rate was 500 Hz. A bandpass filter of 0.53–30 Hz was used at ECG recording and 0–15 Hz was used at SCL recording.

#### Procedure

**FIGURE 1:**
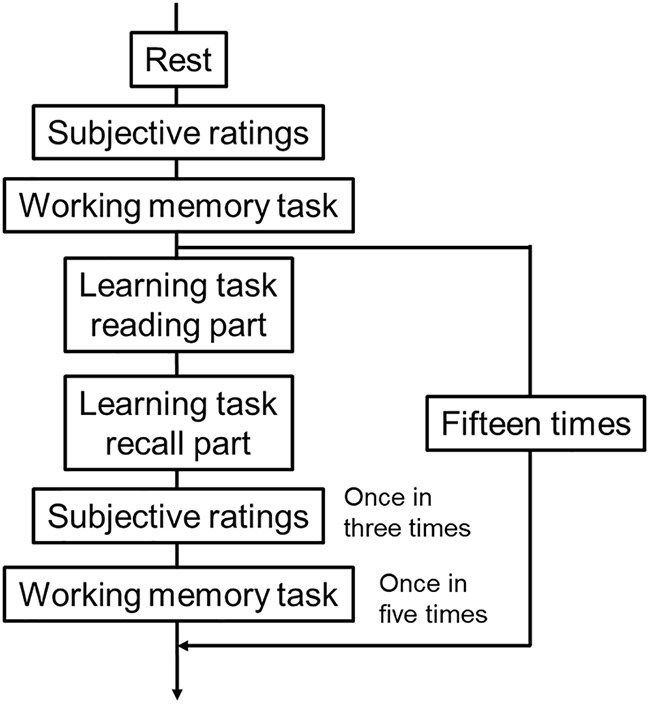
The procedure of this experiment. One set was composed of one rest session, six subjective rating sessions, four working memory sessions, and 15 learning task sessions.

Figure 1 illustrates the procedure of this experiment. This experiment was divided into 2 days. On the first day, the temperature and humidity of the experimental room was set to 18, 22, 25, or 29°C and 50%. They were manipulated by air conditioners FXYFP45M, S56VTAXV-W (Daikin Industries, Ltd.) and the humidifiers HD-9018, HD-243 (Dainichi Co., Ltd.). In the experimental room, participants changed into sweatshirts from their clothes and sat in a chair. In addition, participants were asked not to move their bodies more than necessary in each session to avoid artifacts of physiological data.

In the rest session, participants were asked to gaze at the fixation cross on the display for 120 s. This black fixation cross (visual angle of 2.86° × 2.86° from a viewing distance of 60 cm) was presented at the center of a white background.

After the rest session, participants replied to the subjective rating queries with a keyboard in the subjective rating session. After this, the subjective rating session was presented every third time after the learning task session.

**FIGURE 2:**
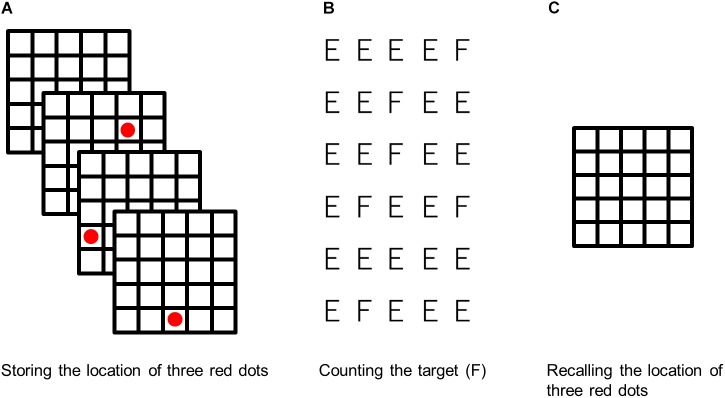
The procedure of the working memory task. Participants were required to **(A)** store the location of three red dots, **(B)** count the target letter, and **(C)** click the location of presentation of each red dot.

In the working memory task session (Figure 2), participants were asked to memorize the location of three red dots that were presented sequentially and randomly in a five-by-five black grid (Figure 2A). The interval between the presentation of the first red dot and the presentation of the grid was set to 1,000 ms, and the duration of the presentation of each red dot was set to 500 ms. After the presentation of three red dots, six-by-five letters (E and F) were presented on the display for 30 s as a delayed task (Figure 2B). Participants were required to count the target letter (F) and reply to the number of the target letter after the presentation of these letters. The set size of the target letters was set to 7 to 9. After the delayed task, the five-by-five black grid was presented again, and participants clicked the location of the presentation of each red dot with a mouse pointer (Figure 2C). This series of working memory tasks was repeated twice; after this, this task was done every fifth time after the learning task session.

**FIGURE 3:**
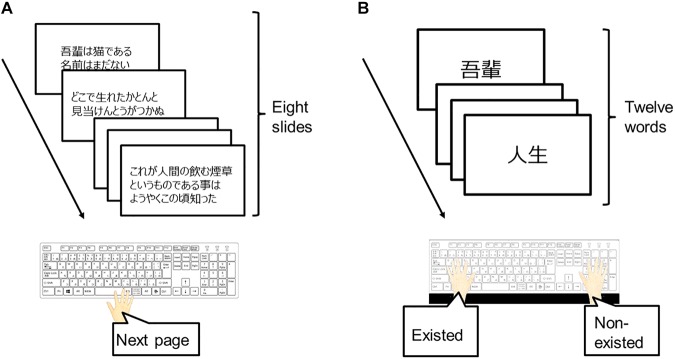
The procedure of the learning task. **(A)** In the reading part, participants were required to read one text at their own pace and memorize the details of this text. **(B)** In the recall part, participants were required to state whether displayed words existed in the text.

The learning task session was composed of a reading part and a recall part. In the reading part (Figure 3A), participants were required to read one text and memorize the details of this text. The one text was divided into eight slides, and these slides were presented sequentially by pressing the space key at one’s own pace. The text was chosen randomly from 60 texts and did not duplicate; in other words, no participant read the same text twice in the experiment. In the recall part (Figure 3B), a keyword, non-keyword, or novel word was presented on the display. Participants were required to press the F key if this word existed in the text (i.e., keyword and non-keyword); otherwise (i.e., novel word), participants were required to press the J key. Twelve words (i.e., keyword: 3; non-keyword: 3; novel word: 6) were presented per session. The inter-stimulus interval was set to 1,000 ms. The order of presentation of words was randomized.

One set was composed of one rest session, six subjective ratings sessions, four working memory sessions, and 15 learning task sessions. After the first set, participants took a rest in the other room that was set to 24°C for 30 min, and the temperature of the experimental room was set to a different temperature from the first block. After resting, participants participated in the second set following the same procedure used in the first set. On the second day, participants participated in third and fourth sets during which the experimental room was set to different temperatures from the first day. The order of temperatures of the experimental room was counterbalanced among participants. Each set took approximately 60 min, and each day took approximately 180 min.

#### Data Analysis

To analyze the learning task data, reading times of the reading part, reaction times, and hit rate of the recall part were calculated. Moreover, these data were averaged across three sessions; i.e., these data were combined to five blocks per one set.

The distances between the presentation of each red dot and each of a participants’ answers were calculated as scores of the working memory task. If a participant clicked the neighboring square of red dot in the grid, the working memory score was increased one point; therefore, this score was zero points when participant correctly clicked all locations of red dots. Moreover, we subtracted the first score (i.e., the score before the learning task session) from subsequent scores (Δworking memory score).

As with the working memory score, subjective ratings before the learning task session were subtracted from subsequent ratings for other measures; i.e., we calculated Δarousal, Δvalence, Δmental demand, Δperformance, Δeffort, Δfrustration, Δoverall mental workload, Δthermal sensation, and Δhumidity sensation.

The EEGLAB toolbox (Delorme and Makeig, 2004) and ERPLAB toolbox (Lopez-Calderon and Luck, 2014) on MATLAB (MathWorks Inc.) were used to analyze the EEG data. Artifacts derived from eye movements and eye blinks were rejected using an automatic EEG artifact detector based on the joint use of spatial and temporal features (ADJUST) of the EEGLAB toolbox (Mognon et al., 2011). After artifact rejection, the EEG data were digitally band-pass filtered at 4–7 Hz (6 dB/octave; order: 5,000) using an IIR Butterworth analog simulation filter. Moreover, this voltage was squared and calculated natural logarithms at the F3 and F4 electrodes to analyze the theta band power as an index of concentration. The ARTiiFACT (Kaufmann et al., 2011) was used to analyze the ECG. R–R intervals were detected from the ECG, and heart rate (HR) and frequency ratios of normalized unit LF/HF were calculated. All physiological data were averaged per three sessions and combined into five blocks. Moreover, we subtracted the data of averaged rest session from these data, i.e., we calculated Δtheta power, ΔHR, ΔLF/HF, ΔSCL, and Δtympanum temperature.

Two-way repeated measures analysis of variance (ANOVA) on reading times in the reading part, reaction times, and hit rate in the recall part, all Δsubjective ratings, and all Δphysiological data in each part of the learning session were conducted with the four conditions (18, 22, 25, and 29°C) and five blocks. Moreover, the Δworking memory score was assessed with two-way repeated measures ANOVA (four conditions and three sessions). These ANOVAs were conducted by applying Greenhouse–Geisser corrections to the degrees of freedom when appropriate (Greenhouse and Geisser, 1959). The effect sizes have been indicated in terms of partial eta squared ($\eta_{\text{p}}^{2}$). *Post hoc* comparisons were made using Shaffer’s modified sequentially rejective multiple test procedure, which extends Bonferroni *t* tests in a stepwise fashion (Shaffer, 1986).

## Results

### Learning Task

**FIGURE 4:**
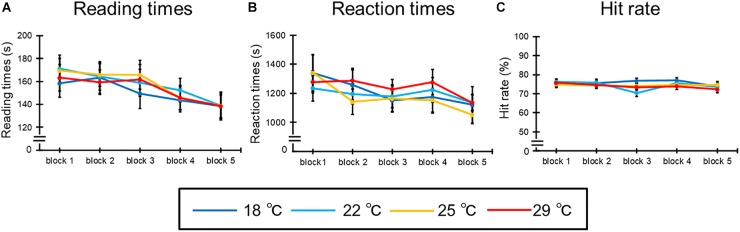
The results of **(A)** reading times, **(B)** reaction times, and **(C)** hit rate for the learning task. The error bar indicates standard error.

Figure 4A shows the mean reading times of all participants in each condition. The ANOVA revealed the main effect of blocks [*F*(4,92) = 21.07, *p* < 0.001, $\eta_{\text{p}}^{2}$ = 0.48]. *Post hoc* comparisons indicated that the reading times of the fourth and fifth blocks were shorter than those of the other blocks; additionally, the reading time of the fifth block was shortest (*p*s < 0.05). However, the main effect of condition [*F*(3,69) = 0.19, *p* = 0.87] and the interaction [*F*(12,276) = 0.64, *p* = 0.68] were not significant.

Figure 4B illustrates the mean RTs of all participants in each condition. The ANOVA for the mean RTs revealed a significant main effect of blocks [*F*(4,92) = 8.28, *p* < 0.001, $\eta_{\text{p}}^{2}$ = 0.26]. *Post hoc* comparisons indicated that the reaction times of the fifth block were shorter than those of the other blocks (*p*s < 0.05). However, the main effects of condition [*F*(3,69) = 0.32, *p* = 0.76] and interaction [*F*(12,276) = 1.01, *p* = 0.41] were not significant. Moreover, Figure 4C shows the mean hit rates of all participants in each condition. The ANOVA revealed that all main effects [condition: *F*(3,69) = 0.19, *p* = 0.87; blocks: *F*(4,92) = 1.17, *p* = 0.33] and interactions [*F*(12,276) = 0.64, *p* = 0.68] were not significant.

#### Working Memory Task

**FIGURE 5:**
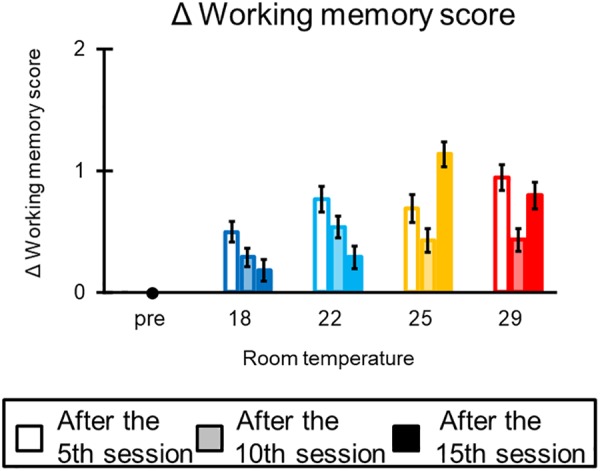
The results of the working memory task. The error bar indicates standard error. A high value of the working memory score means poor performance for this task.

Figure 5 illustrates the mean working memory score of all participants in each condition. The ANOVA for the mean Δworking memory score indicated that all main effects [condition: *F*(3,69) = 0.79, *p* = 0.49; block: *F*(2,46) = 0.63, *p* = 0.53] and interactions [*F*(6,138) = 0.48, *p* = 0.78] were not significant.

### Subjective Rating

**FIGURE 6:**
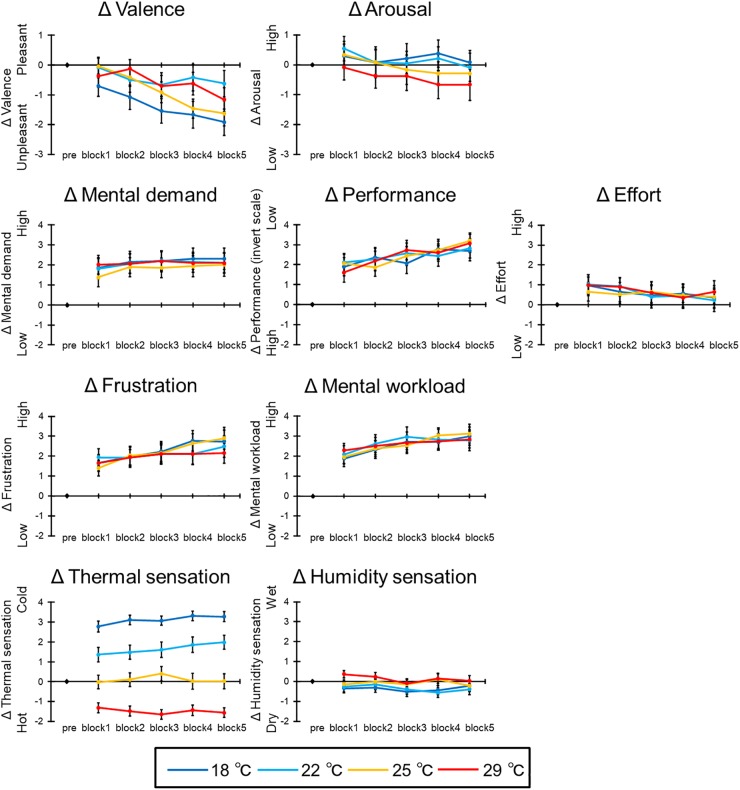
The results of Δsubjective ratings. The error bar indicates standard error.

Figure 6 illustrates the mean subjective ratings of all participants in each condition, and Table 1 shows the ANOVA results for these subjective ratings.

**TABLE 1:** The results of ANOVA for Δsubjective ratings.

	**Room temperature**			**Block**			**Interaction**		
Item	***df* = (3, 69)**			***df* = (4, 92)**			***df* = (12, 276)**		
	***F***	***p***	**$\eta_{\text{p}}^{2}$**	***F***	***p***	**$\eta_{\text{p}}^{2}$**	***F***	***p***	**$\eta_{\text{p}}^{2}$**
**Valence and arousal**									
ΔValence	2.11	0.13	0.08	7.25	< 0.001	0.43	1.95	0.07	0.08
ΔArousal	1.19	0.32	0.05	4.84	0.004	0.17	0.62	0.70	0.03
**NASA-TLX**									
ΔMental demand	0.94	0.42	0.04	3.77	0.02	0.14	0.55	0.77	0.02
ΔPerformance	0.05	0.98	0.01	16.11	< 0.001	0.41	1.46	0.18	0.06
ΔEffort	0.26	0.81	0.01	4.15	0.02	0.15	0.89	0.51	0.04
ΔFrustration	0.22	0.86	0.01	8.55	< 0.001	0.30	1.38	0.24	0.06
ΔMental workload	0.12	0.92	0.01	11.28	< 0.001	0.33	1.09	0.37	0.05
**Thermal sensation and humidity sensation**									
ΔThermal sensation	171.93	< 0.001	0.88	2.82	< 0.05	0.11	2.51	0.02	0.10
ΔHumidity sensation	2.47	0.10	0.10	2.55	0.07	0.10	1.11	0.36	0.05

#### Valence and Arousal

In the Δvalence, the main effect of blocks was significant [*F*(4,92) = 7.25, *p* < 0.001, $\eta_{\text{p}}^{2}$ = 0.43]. *Post hoc* comparisons indicated that the point of valence after the third block was lower than (i.e., unpleasant) before the second block (*p*s < 0.05). The main effect of condition [*F*(3,69) = 2.11, *p* = 0.13] and the interaction [*F*(12,276) = 1.95, *p* = 0.07] were not significant. Moreover, the main effect of blocks in the Δarousal was significant [*F*(4,92) = 4.84, *p* = 0.004, $\eta_{\text{p}}^{2}$ = 0.17]. *Post hoc* comparisons indicated that the point of arousal under the fifth block was lower than (i.e., low arousal) the first block (*p* < 0.05). The main effect of condition [*F*(3,69) = 1.19, *p* = 0.32] and the interaction [*F*(12,276) = 0.62, *p* = 0.70] were not significant.

#### NASA-TLX

In the Δmental demand, the main effect of blocks was significant [*F*(4,92) = 3.77, *p* = 0.02, $\eta_{\text{p}}^{2}$ = 0.14]; however, *post hoc* comparisons were not significant. Additionally, the main effect of condition [*F*(3,69) = 0.94, *p* = 0.42] and the interaction [*F*(12,276) = 0.55, *p* = 0.77] were not significant. In the Δperformance, the main effect of blocks was significant [*F*(4,92) = 16.11, *p* < 0.001, $\eta_{\text{p}}^{2}$ = 0.41]. *Post hoc* comparisons indicated that the subjective performance after the third block was lower than before the second block (*p*s < 0.05). The main effect of condition [*F*(3,69) = 0.05, *p* = 0.98] and the interaction [*F*(12,276) = 1.46, *p* = 0.18] were not significant. In the Δeffort, the main effect of blocks was significant [*F*(4,92) = 4.15, *p* = 0.02, $\eta_{\text{p}}^{2}$ = 0.15]; however, *post hoc* comparisons were not significant. Additionally, the main effect of condition [*F*(3,69) = 0.26, *p* = 0.81] and the interaction [*F*(12,276) = 0.89, *p* = 0.51] were not significant. In the Δfrustration, the main effect of blocks was significant [*F*(4,92) = 8.55, *p* < 0.001, $\eta_{\text{p}}^{2}$ = 0.30]. *Post hoc* comparisons indicated that the frustration after the fourth block was larger than in the first block; additionally, the fifth block was larger than the second block (*p*s < 0.05). The main effect of condition [*F*(3,69) = 0.22, *p* = 0.86] and the interaction [*F*(12,276) = 1.38, *p* = 0.24] were not significant. In the Δoverall mental workload, the main effect of blocks was significant [*F*(4,92) = 11.28, *p* < 0.001, $\eta_{\text{p}}^{2}$ = 0.33]. *Post hoc* comparisons indicated that the overall mental workload after the third block was larger than before the second block; additionally, those after the fourth block were larger than in the second block (*p*s < 0.05). The main effect of condition [*F*(3,69) = 0.12, *p* = 0.92] and the interaction [*F*(12,276) = 1.09, *p* = 0.37] were not significant.

#### Thermal Sensation and Humidity Sensation

In the Δthermal sensation, the interaction was significant [*F*(12,276) = 2.51, *p* = 0.02, $\eta_{\text{p}}^{2}$ = 0.10]. *Post hoc* comparisons indicated that the 18°C condition was evaluated as colder than the other thermal conditions under the all blocks (*p*s < 0.05). In addition, the 22°C condition was evaluated as colder than the 25 and 29°C conditions, and the 25°C condition was evaluated as colder than the 29°C condition (*p*s < 0.05). Moreover, the main effect of condition was significant [*F*(3,69) = 171.9, *p* < 0.001, $\eta_{\text{p}}^{2}$ = 0.88]. *Post hoc* comparisons indicated the same result of the interaction (*p*s < 0.05). The main effect of blocks was significant [*F*(4,92) = 2.82, *p* < 0.05, $\eta_{\text{p}}^{2}$ = 0.11]; however, *post hoc* comparisons were not significant. In the Δhumidity sensation, all main effects [condition: *F*(3,69) = 2.47, *p* = 0.10; block: *F*(4,92) = 2.55, *p* = 0.07] and the interaction [*F*(12,276) = 1.11, *p* = 0.36] were not significant.

### Physiological Data

#### Electroencephalogram

**FIGURE 7:**
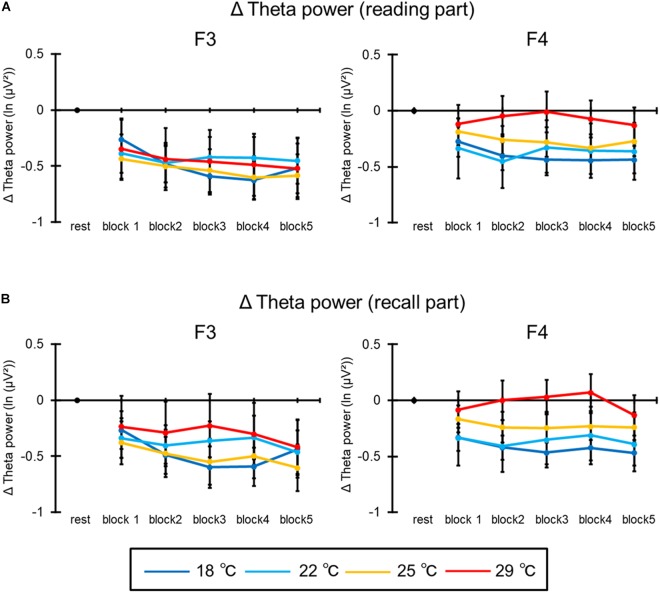
The results of Δtheta power recorded by the F3 and F4 electrodes in **(A)** the reading part and **(B)** the recall part. The error bar indicates standard error.

Figure 7A shows the mean theta power of all participants in the reading part of the learning session. In the Δtheta power recorded by F3, the ANOVA revealed the main effect of blocks [*F*(4,92) = 5.73, *p* = 0.006, $\eta_{\text{p}}^{2}$ = 0.20]. *Post hoc* comparisons indicated that the Δtheta power of the second and fourth blocks were smaller than the first block. The main effect of condition [*F*(3,69) = 0.07, *p* = 0.97] and the interaction [*F*(12,276) = 1.23, *p* = 0.30] were not significant. Moreover, all main effects [condition: *F*(3,69) = 1.20, *p* = 0.34; block: *F*(4,92) = 1.88, *p* = 0.17] and the interaction [*F*(12,276) = 1.89, *p* = 0.10] were not significant in the Δtheta power recorded by F4.

Figure 7B illustrates the mean theta power of all participants in the recall part of the learning session. In the Δtheta power recorded by F3, the ANOVA revealed the main effect of blocks [*F*(4,92) = 3.34, *p* = 0.04, $\eta_{\text{p}}^{2}$ = 0.13]; however, *post hoc* comparisons were not significant. Additionally, the main effect of condition [condition: *F*(3,69) = 0.28, *p* = 0.82] and the interaction [*F*(12,276) = 1.31, *p* = 0.28] were not significant. Moreover, all main effects [condition: *F*(3,69) = 1.68, *p* = 0.18; block: *F*(4,92) = 1.33, *p* = 0.26] and the interaction [*F*(12,276) = 1.12, *p* = 0.34] were not significant in the Δtheta power recorded by F4.

#### Heart Rate

**FIGURE 8:**
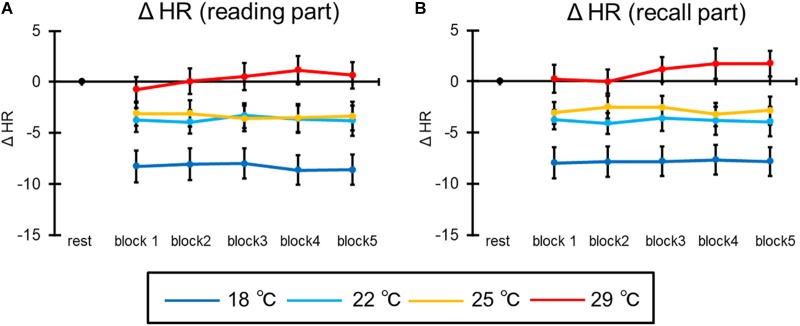
The results of ΔHR in **(A)** the reading part and **(B)** the recall part. The error bar indicates standard error.

Figure 8A shows the mean HR of all participants in the reading part of the learning session. The ANOVA revealed the main effect of condition [*F*(3,69) = 12.55, *p* < 0.001, $\eta_{\text{p}}^{2}$ = 0.35]. *Post hoc* comparisons indicated that the ΔHR of the 18°C condition was smaller than in the other conditions (*p*s < 0.05). The main effect of blocks [*F*(4,92) = 0.45, *p* = 0.62] and the interaction [*F*(12,276) = 1.26, *p* = 0.28] were not significant.

Moreover, Figure 8B illustrates the mean HR of all participants in the recall part of the learning session. The ANOVA revealed the main effect of condition [*F*(3,69) = 12.93, *p* < 0.001, $\eta_{\text{p}}^{2}$ = 0.36]. *Post hoc* comparisons indicated that the ΔHR of the 18°C condition was smaller than in the other conditions. In addition, the ΔHR of the 22°C condition was smaller than in the other conditions (*p*s < 0.05). The main effect of blocks [*F*(4,92) = 0.74, *p* = 0.47] and the interaction [*F*(12,276) = 0.93, *p* = 0.48] were not significant.

#### Low Frequency to High Frequency

**FIGURE 9:**
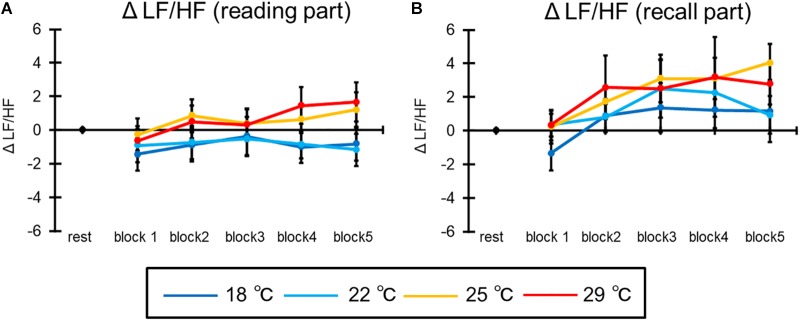
The results of ΔLF/HF in **(A)** the reading part and **(B)** the recall part. The error bar indicates standard error.

Figure 9A shows the mean LF/HF of all participants in the reading part of the learning session. The ANOVA revealed the interaction [*F*(12,276) = 2.39, *p* < 0.05, $\eta_{\text{p}}^{2}$ = 0.09]. *Post hoc* comparisons indicated that the ΔLF/HF was higher at 29°C than at 18°C and 22°C in the second block, and at 25 and 29°C than at 18 and 22°C after the fourth block (*p*s < 0.05). In addition, ΔLF/HF was higher at the second and fifth blocks than at the first block in the 25°C, and at the second, fourth, and fifth blocks than at first block in the 29°C. Moreover, the main effects of condition [*F*(3, 69) = 9.51, *p* < 0.001, $\eta_{\text{p}}^{2}$ = 0.29] and block [*F*(4,92) = 7.32, *p* < 0.001, $\eta_{\text{p}}^{2}$ = 0.29] were significant. *Post hoc* comparisons indicated that the ΔLF/HF was higher at 25 and 29°C than at 18 and 22°C, and at the after second block than at the first block (*p*s < 0.05).

Furthermore, Figure 9B illustrates the mean LF/HF of all participants in the recall part of the learning session. The ANOVA revealed the main effect of blocks [*F*(4,92) = 3.10, *p* < 0.05, $\eta_{\text{p}}^{2}$ = 0.12]. *Post hoc* comparisons indicated that the ΔLF/HF was higher at the second block than at the first block (*p*s < 0.05). The main effect of condition [*F*(3,69) = 1.45, *p* = 0.24] and the interaction [*F*(12,276) = 0.32, *p* = 0.92] were not significant.

#### Skin Conductance Level

**FIGURE 10:**
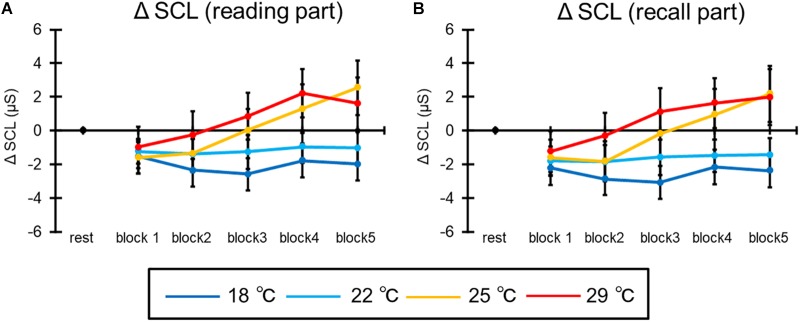
The results of ΔSCL in **(A)** the reading part and **(B)** the recall part. The error bar indicates standard error.

Figure 10A shows the mean SCL of all participants in the reading part of the learning session. The ANOVA revealed the interaction [*F*(12,276) = 4.75, *p* = 0.003, $\eta_{\text{p}}^{2}$ = 0.17]. *Post hoc* comparisons indicated that the ΔSCL was higher at 25 and 29°C than at 18°C in the third and fourth blocks, and at 25 and 29°C than at 18 and 22°C in the fifth block (*p*s < 0.05). In addition, ΔSCL was higher at the fifth block than at the first and third blocks, and at the fourth and fifth blocks than at the second block in the 25°C condition (*p*s < 0.05). In the 29°C, ΔSCL was higher at the third and fifth blocks than at the first block, and at the fourth block than at the second block (*p*s < 0.05). Moreover, the main effect of blocks was significant [*F*(4,92) = 10.24, *p* < 0.001, $\eta_{\text{p}}^{2}$ = 0.31]. *Post hoc* comparisons indicated that the ΔSCL was higher at the fourth and fifth blocks than at the other blocks (*p*s < 0.05). The main effect of condition was not significant [*F*(3,69) = 2.93, *p* = 0.06].

Furthermore, Figure 10B illustrates the mean SCL of all participants in the recall part of the learning session. The ANOVA revealed the interaction [*F*(12,276) = 3.85, *p* = 0.007, $\eta_{\text{p}}^{2}$ = 0.14]. *Post hoc* comparisons indicated that the ΔSCL was higher at 25 and 29°C than at 18°C in the third and fourth blocks, and at 25 and 29°C than at 18°C, and at 25°C than 22°C in the fifth block (*p*s < 0.05). In addition, ΔSCL was higher at after the third block than at the second block in the 25°C condition (*p*s < 0.05). In the 29°C condition, ΔSCL was higher after the third block than at the first block, and at the fourth block than at the second block (*p*s < 0.05). Moreover, the main effects of condition [*F*(3,69) = 4.33, *p* = 0.02, $\eta_{\text{p}}^{2}$ = 0.16] and block [*F*(4,92) = 8.42, *p* = 0.002, $\eta_{\text{p}}^{2}$ = 0.27] were significant. *Post hoc* comparisons indicated that the ΔSCL was higher at 25 and 29°C than at 18°C, and after the third block than at the second block (*p*s < 0.05).

#### Tympanum Temperature

**FIGURE 11:**
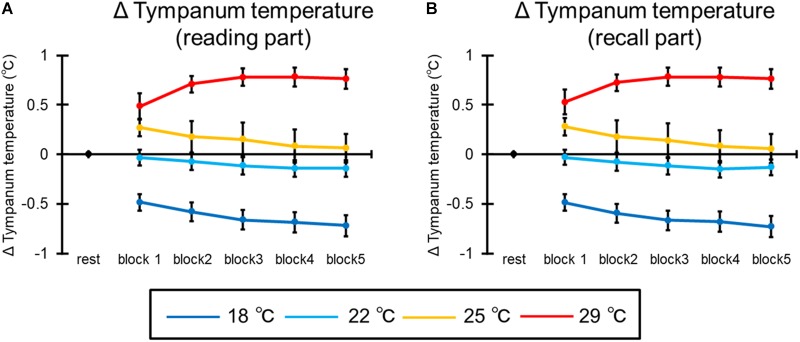
The results of Δtympanum temperature in **(A)** the reading part and **(B)** the recall part. The error bar indicates standard error.

Figure 11A shows the mean tympanum temperature of all participants in the reading part of the learning session. The ANOVA revealed the interaction [*F*(12,276) = 4.96, *p* = 0.006, $\eta_{\text{p}}^{2}$ = 0.18]. *Post hoc* comparisons indicated that the Δtympanum temperature was higher at 22, 25, and 29°C than 18°C, and at 25 and 29°C than 22°C in the first block (*p*s < 0.05). In addition, the Δtympanum temperature was higher at 22, 25, and 29°C than 18°C, and at 29°C than 22 and 25°C after the second block (*p*s < 0.05). In the 18°C, Δtympanum temperature was higher at the first block than at after the second block, and at the second block than after the third block, and at the third block than at the fifth block (*p*s < 0.05). Moreover, the main effect of condition was significant [*F*(3,69) = 38.26, *p* < 0.001, $\eta_{\text{p}}^{2}$ = 0.62]. *Post hoc* comparisons indicated that the Δtympanum temperature was higher at 22, 25, and 29°C than 18°C, and at 29°C than 22 and 25°C (*p*s < 0.05). The main effect of block was not significant [*F*(4,92) = 1.56, *p* = 0.23].

Furthermore, Figure 11B illustrates the mean tympanum temperature of all participants in the recall part of the learning session. The ANOVA revealed the interaction [*F*(12,276) = 4.93, *p* = 0.006, $\eta_{\text{p}}^{2}$ = 0.18]. *Post hoc* comparisons indicated that the Δtympanum temperature was higher at 22, 25, and 29°C than 18°C, and at 25 and 29°C than 22°C in the first block (*p*s < 0.05). In addition, the Δtympanum temperature was higher at 22, 25, and 29°C than 18°C, and at 29°C than 22 and 25°C after the second block (*p*s < 0.05). In the 18°C, Δtympanum temperature was higher at the first block than at after the second block, at the second block than after the third block, and at the third block than at the fifth block (*p*s < 0.05). Moreover, the main effect of condition was significant [*F*(3,69) = 38.79, *p* < 0.001, $\eta_{\text{p}}^{2}$ = 0.63]. *Post hoc* comparisons indicated that the Δtympanum temperature was higher at 22, 25, and 29°C than 18°C, and at 29°C than 22 and 25°C (*p*s < 0.05). The main effect of block was not significant [*F*(4,92) = 2.18, *p* = 0.15].

## Discussion

The present study aimed to investigate whether the thermal environment influences task performance over time and whether the relationship between task performance and thermal environment influences psychological states of participants. For this purpose, we developed a learning task that allowed us to continuously evaluate the change in performance over time, and to compare the task performance, subjective rating for mental workload, working memory score, and physiological index during the task between different room temperature conditions.

Our results showed that mean reading times and RTs were facilitated over time in all conditions. Moreover, the hit rate was kept over 70% throughout the task in all conditions. In addition, working memory score did not decrease through the task in all conditions and did not differ between conditions. These results suggest that the qualitative learning efficiency (i.e., hit rate) was maintained during the task and temporal learning efficiency of the input and output of the information was improved with progress on the task.

In addition, our results showed that the theta power of EEG recorded by the left frontal electrode decreased with time in the reading part. This decrease does not imply an impairment of task performance, because the hit rate was maintained during the task and the mean reading time and RTs were facilitated by progress on the task. The frontal theta power reflects cognitive control and allocation of cognitive resources (e.g., Cavanagh and Frank, 2014). Although previous studies reported that the frontal theta power increased with task difficulty and the level of workload (e.g., Gevins et al., 1997; Miklody et al., 2017; Di Flumeri et al., 2018), other studies reported that this power decreased with facilitation of the task performance when the task difficulty was fixed (e.g., Pathania et al., 2019). Decreased power with facilitated task performance suggested that excessive cognitive resources were needed to perform the task at the beginning of the task; however, excessive cognitive resources were suppressed and appropriate cognitive resource were allocated over time, resulting in the task performance being maintained. In the present study, the qualitative learning efficiency was maintained during the task and learning efficiency was facilitated by progress on the task. Therefore, it is thought that excessive cognitive resources were suppressed over time and that the cognitive processing of the learning was streamlined in the present study. By contrast, the theta power recorded by the right frontal electrode did not decrease during the task. In the present study, the learning task was a memory task in which participants were required to memorize details of a text in the reading part. It is reported that the fluctuation of theta power occurs with left hemisphere superiority in the memory task which measured the EEG (e.g., Bastiaansen et al., 2005). Therefore, it is thought that the difference in theta power between the left and right electrodes occurred due to characteristics of the task in this study. Moreover, the theta power did not differ in the recall part. A previous study reported that frontal theta power increased with effort required for recall and that this increase occurred between 2,500 and 3,500 ms after onset of the presentation for recall (e.g., Khader and Rösler, 2011). In the present study, participants responded in less than 1,400 ms in the recall part and the hit rate was kept at over 70% throughout the task. Therefore, it is possible that fluctuation of the theta power did not appear in the recall part.

Taken together, the fixed room temperature does not impair learning efficiency, and learning efficiency is facilitated over time in all conditions. However, the subjective frustration and mental workload increased, and subjective valence, arousal, and performance decreased with time. These results indicate that the subjective mental workload increases, even if learning efficiency is facilitated. Task performance seems to be improved by experiencing many trials in spite of discomfort induced by a high room temperature.

In addition, the other physiological index was influenced by room temperature. The tympanum temperature rose and HR increased with increases in room temperature. It is thought that this result was a physiological response related to adapting to the room temperature and that this adaptation was finished at the beginning of the task because neither tympanum temperature nor HR increased with time. The blood vessels dilate to facilitate heat radiation from the skin when the room temperature increases. In this situation, blood output per heartbeat and blood pressure decrease. Thus, to excrete the same amount of blood as usual, it is necessary to increase the HR by activating the sympathetic nervous system (e.g., Ishibashi and Yasukouchi, 1999). It is thought that results of tympanum temperature and HR reflect this physiological response related to adaptation to room temperature and that this adaptation was finished at the beginning of the task.

Furthermore, LF/HF increased with time and room temperature (i.e., 25 and 29°C) in the reading part. It is thought that increased LF/HF at the beginning of the task reflects the heat radiation like the HR does. However, the increase at the end of the task cannot be explained by this heat radiation, because, according to the results of tympanum temperature and HR, the adaptation to the room temperature was finished at the beginning of the task. Therefore, it is possible that this increase reflects not the heat radiation but the mental workload generated by repeating a task (e.g., Berntson and Cacioppo, 2004). By contrast, LF/HF did not increase in the recall part. LF/HF needs 20–30 s to change per period because of the frequency (e.g., Berntson and Cacioppo, 2004; LF: 0.04–0.15 Hz; HF: 0.15–0.4 Hz). In this study, the total time in the recall part per block was approximately under the 30 s. Therefore, it is possible that this part was finished before the beginning of an increase in the LF/HF.

Finally, SCL increased with time in each part. In particular, it was remarkable in the relatively warm room (i.e., 25 and 29°C) at the end of task. It is thought that this increase reflects the mental workload generated by repeating a task (e.g., Reimer and Mehler, 2011; Foy and Chapman, 2018), like the result for the LF/HF in the reading part.

In summary, as time elapsed, excessive concentration on the task was suppressed and learning efficiency was facilitated while the warm environment increased the subjective mental workload. This increased subjective stress cannot be evaluated by the task performance. The physiological response for mental workload is an adaptive response to activate the body, and we can perform the task in each situation using this activation (e.g., Cannon, 1929). In other words, we can perform the task as well as usual by activating the body even in the case where task execution is difficult. Therefore, our results showing that task performance is maintained or improved through trials are due to this activation. And the mental workload increases in relation to this activation. From this point of view, maintaining task performance in a severe environmental condition is consequently accompanied by a stress response. Long-term continuous work with a high mental workload increases health risks (e.g., Selye, 1956). Therefore, it is necessary to decrease the mental workload while task performance is maintained in various environments. Since a warmer environment causes an increased mental workload with progress on the task, the mental workload can be adjusted to a suitable level by controlling the thermal environment during progress on the task. Our results propose that it is necessary to consider not only the task performance but also the mental workload of the student and worker in order to configure an appropriate temperature for work and study.

Several points to examine remain for future studies. In the present study, we defined learning efficiency as a function based on various cognitive activities and we examined the relationship between learning efficiency and room temperature. Even though the input and output of information are commonly important for various types of work, a previous study reported that the effect of room temperature differently affects performance on some cognitive tasks (e.g., Lan et al., 2009). In addition, implicit stress might increase as in our experiment, even if the performance of cognitive tasks is not impaired. In future research, relationships between room temperature, various cognitive activities, and mental workload should be examined. Moreover, in our study, participants’ clothes were fixed in each room temperature. It might be possible to reduce stress if participants can freely adjust the amount of their clothing. In addition, previous studies reported different stress responses between manageable stress situations and others (e.g., Obrist et al., 1978). Therefore, adjusting one’s own clothes might function not only to adjust body temperature but also to actively cope with stress generated by a room’s temperature. It is necessary to examine the stress response when active coping is executed at each room temperature in the future. Furthermore, it is necessary to examine the factors of age and sex. In the present study, participants were 20–24 years old, assuming the age of students and workers. However, the age-range of students and workers is wide (e.g., Niemelä et al., 2002; Tanabe et al., 2009; Haverinen-Shaughnessy and Shaughnessy, 2015; Wargocki et al., 2019), and the factor of age relates to differences in working memory performance (e.g., Wild-Wall et al., 2011). Thus, it is possible that factor of age influences the relationship between room temperature and mental workload, and taking together these studies and our results, the effect of a warmer environment might vary with age. In addition, previous research reported that the influence of room temperature on cognitive performance varied between males and females (e.g., Chang and Kajackaite, 2019). In future research, the relationship among room temperature and factors of age and sex should be examined, and, to conduct these additional analyses, it will be necessary to record the data from a larger sample.

## Conclusion

The present study showed that a warm environment increased subjective mental workload even if excessive concentration on the task was suppressed and learning efficiency was facilitated. This increased subjective stress cannot be evaluated by task performance; it was first revealed by examining task performance, subjective assessment, and physiological data comprehensively. Our results propose that it is necessary to consider not only the task performance but also the mental workload of students and workers in order to configure a thermal environment appropriate for work and study.

## Data Availability Statement

The datasets generated for this study will not be made publicly available because these data contains personal information. Requests to access the datasets should be directed to corresponding author.

## Ethics Statement

The studies involving human participants were reviewed and approved by the Behavioral Research Ethics Committee of the Osaka University School of Human Sciences. The patients/participants provided their written informed consent to participate in this study.

## Author Contributions

TK and KS contributed to the conception and design of the study. TK, NT, YN, and HK contributed to the data acquisition. TK wrote the first draft of the manuscript. All authors contributed to the statistical analysis, discussing, revising, and reading the manuscript, and finally approved the submitted version.

## Conflict of Interest

HK was employed by the company Daikin Industries, Ltd.

The remaining authors declare that the research was conducted in the absence of any commercial or financial relationships that could be construed as a potential conflict of interest.
